# It Costs to Be Clean and Fit: Energetics of Comfort Behavior in Breeding-Fasting Penguins

**DOI:** 10.1371/journal.pone.0021110

**Published:** 2011-07-19

**Authors:** Vincent A. Viblanc, Adeline Mathien, Claire Saraux, Vanessa M. Viera, René Groscolas

**Affiliations:** 1 Université de Strasbourg, Institut Pluridisciplinaire Hubert Curien, Département Ecologie, Physiologie et Ethologie, Strasbourg, France; 2 Centre National de la Recherche Scientifique, Unité Mixte de Recherche 7178, Strasbourg, France; 3 AgroParisTech, Ecole Nationale du Génie Rural, des Eaux et des Forêts, Paris, France; 4 Département de biologie et Centre d'études nordiques, Université Laval, Québec, Canada; Roehampton University, United Kingdom

## Abstract

**Background:**

Birds may allocate a significant part of time to comfort behavior (e.g., preening, stretching, shaking, etc.) in order to eliminate parasites, maintain plumage integrity, and possibly reduce muscular ankylosis. Understanding the adaptive value of comfort behavior would benefit from knowledge on the energy costs animals are willing to pay to maintain it, particularly under situations of energy constraints, e.g., during fasting. We determined time and energy devoted to comfort activities in freely breeding king penguins (*Aptenodytes patagonicus*), seabirds known to fast for up to one month during incubation shifts ashore.

**Methodology/Principal Findings:**

A time budget was estimated from focal and scan sampling field observations and the energy cost of comfort activities was calculated from the associated increase in heart rate (HR) during comfort episodes, using previously determined equations relating HR to energy expenditure. We show that incubating birds spent 22% of their daily time budget in comfort behavior (with no differences between day and night) mainly devoted to preening (73%) and head/body shaking (16%). During comfort behavior, energy expenditure averaged 1.24 times resting metabolic rate (RMR) and the corresponding energy cost (i.e., energy expended in excess to RMR) was 58 kJ/hr. Energy expenditure varied greatly among various types of comfort behavior, ranging from 1.03 (yawning) to 1.78 (stretching) times RMR. Comfort behavior contributed 8.8–9.3% to total daily energy expenditure and 69.4–73.5% to energy expended daily for activity. About half of this energy was expended caring for plumage.

**Conclusion/Significance:**

This study is the first to estimate the contribution of comfort behavior to overall energy budget in a free-living animal. It shows that although breeding on a tight energy budget, king penguins devote a substantial amount of time and energy to comfort behavior. Such findings underline the importance of comfort behavior for the fitness of colonial seabirds.

## Introduction

Maintenance behaviors (i.e. allo- and autogrooming, allo- and autopreening, bathing, scratching, stretching, etc.) serve a variety of purposes and are widespread throughout the animal kingdom (e.g. in mammals [1]–[3], in birds [4]–[6], in fish [7], in crustaceans [8], and in insects [9]–[10]). Studies that have considered the adaptive significance of maintenance behaviors (referred to as comfort behavior in birds [11]) have suggested both proximate (i.e. bodily) and more ultimate (i.e. social) functions such as the maintenance of good corporeal condition (e.g. parasite control, thermal insulation or muscle condition [2], [5], [12]–[14]) or the maintenance of sexual ornaments [6], [15]–[16]. Maintenance behaviors have also been suggested to be facilitated by social contexts [17], and accredited to play a role in social relationships [3], [18]–[19].

In birds, comfort behavior is usually referred to as a set of activities concerned with the care of the integument and the maintenance of a functional body structure, i.e. by increasing proprioceptive sensitivity and circulation in the muscles for instance [4]–[5], [11]. Several studies have previously shown that birds spend a substantial amount of time in comfort behavior. Indeed, a meta-analysis over 62 different avian species, revealed that birds devoted 9.2% of their daily time budgets to comfort activities [20] (92.6% of which was preening), and figures close to 15% have been reported in several species (15% in gulls [21], 14% in Japanese quail [22], 14.9% in peacocks [6]). Obviously, the time devoted to comfort behavior must trade with that devoted to other activities, which could incur some costs, including indirect energy costs. For example, individuals allocating a higher proportion of time into comfort may face a reduction in resting time, decreased vigilance towards predators, and decreased foraging time. The temporal trade-off dilemma is well illustrated by Walther and Clayton's study [15] who found that, when looking for maintenance times in ornamental and non-ornamental species, wild birds only spent 8.7% of their time on maintenance behaviors whereas captive individuals spent almost twice as much, i.e. 15.8% (see [15]). Captive birds may indeed devote a greater amount of time to comfort behavior, as food is usually provided *ad libitum* in a safe environment, and the amount of time spent foraging or in vigilance may be decreased.

Because of corresponding physical activity, comfort behavior may also incur direct energy costs which may substantially impact overall energy budget. As natural selection is thought to drive the evolution of animal behaviors whenever their benefits outweigh their costs (leading to behavioral strategies that appear differentially adaptive and that ultimately increase individual fitness [23]), estimating the energy costs directly associated with comfort behavior might help in understanding how adaptive strategies could evolve in regards of energy allocation and trade-offs. Such estimates would be particularly informative for species that rely on limited energy supplies for part of their life cycle, e.g. long-term fasters. Indeed, in those species the effective management of energy stores could well mean the difference between survival and death, breeding success and failure. To date, few studies have considered energy costs related to comfort behaviors [2], [24] and those that have done so determined the animal's energy expenditure in response to parasitic infestation rather than the energy expenditure due to comfort *per se*. However, one could presume that high parasite loads may impose energy costs asides those related to grooming. One reason that could well explain the lack of data on the energy costs of specific behaviors, and on the contribution of comfort behavior to overall energy budget, might have to do with methodological issues. Classical methods used to monitor energy expenditure (EE) such as stable isotopes or respirometry, are either not adapted to measure the energetics of specific behaviors (but see [25]), nor readily transposable to field monitoring. Although the doubly labeled water technique (DLW) is relatively simple to use in the field and offers reasonably accurate measures of EE, this method only yields an average estimate over the duration of the experiment. Thus, whereas measuring EE relating to specific activities using DLW may be possible under controlled conditions [25], obtaining those estimates in free-living field conditions and for birds alternating different types of activities of relatively short duration is not possible. On the other hand, determining the contribution of one behavior to energy budget requires an accurate estimate of the time devoted daily to this behavior, which is possible only for animals living in the open and thus easily observable.

In this study, we consider the energetics of comfort behavior under a natural context using breeding-fasting king penguins (*Aptenodytes patagonicus*) as a model. King penguins are long-lived, semi-altricial seabirds that reproduce in vast colonies of several thousands of pairs on beaches of the subantarctic islands [26]. During egg-incubation, which lasts on average 53 days [27], parents take turns to incubate the single egg on their feet, undergoing prolonged periods of fasting ashore while the partner is foraging at sea. The first incubation shift is the longest observed in the species and typically lasts for approximately one month [28]. Subsequent shifts last around 15 days, during which the incubating parents rely mainly on fat stores built up during the previous foraging trips to sustain their metabolism [29]. The important energy constraint of such a reproductive pattern is well illustrated by the fact that parents, because of the critical depletion of their energy stores, sometimes abandon the egg or young chick in order to go and re-feed at sea, before the return of their partner [30]–[31]. In a context where energy savings appear as such a critical issue, the previous finding that incubating male king penguins may devote a substantial part of daily time-budget to comfort behavior [32] might seem somewhat paradoxical even for professional fasters. Such a finding might then be explained by two alternative hypotheses. First, if comfort behavior was not energetically costly and did not trade with other time-consuming behaviors such as foraging (given that birds are incubating and fasting), spending a substantial amount of time in comfort may be the mere consequence of penguins having actually no major time constraints while breeding ashore. Alternately, if comfort behavior was energetically costly, this would indicate that when breeding, penguins are faced with important constraints (such as those related to parasite load or muscular ankylosis), and should pay the energy cost in order to keep in good physical condition, including in anticipation of subsequent foraging trips at sea.

To discriminate between these two hypotheses, we investigated the time and energy budget of comfort behavior in king penguins breeding ashore using heart rate (HR) as a proxy of energy expenditure [33], [34]. We determined the time and energy allocated both to global (i.e. overall) and specific comfort behaviors (e.g. preening, stretching, and shaking). This allowed us to calculate the contribution of comfort behavior to daily energy expenditure, and to suggest the very first estimates of the cost of comfort activities allocated to plumage cares *vs.* non plumage-related comfort behavior in a colonial seabird.

## Methods

### Ethics Statement

Animals in this study were cared for in accordance with the guidelines of the Ethical Committee of the French Polar Institute (Institut Paul Emile Victor – IPEV). All procedures employed during the field work were approved by the committee and comply with current French laws. Authorizations to enter the breeding colony (permit N° 2006-64 issued on November 4, 2006; and permit N° 2007-148 issued on October 24, 2007) and handle birds (permit N° 2006-73 issued on November 6, 2006; and permit 2007-143 issued on October 24, 2007) were delivered by Terres Australes et Antarctiques Françaises. Copies of permits are available upon request. During field procedures, animals were hooded in order to keep them calm and reduce the disturbance to neighboring birds. Manipulations lasted between 5 and 10 min and never resulted in egg or chick abandonment. HR logger packages weighed less than 1% of adult body mass and were installed in a dorsal midline position to prevent hindering movements of the birds. Flipper bands were removed at the end of the study.

### Field Procedure

This study was carried out on Possession Island, Crozet Archipelago (46°25′S, 51°45′E), in the breeding colony of ‘La Baie du Marin’ which is host to over 16.000 pairs of king penguins [35]. During two consecutive breeding seasons (November–March), from 2006 to 2008, a total of 206 incubating and brooding adults were marked using a non-permanent animal dye (Porcimark®, Kruuse, Germany) and/or flipper banded for identification during field observations. Part of the animals (N = 191) was sexed from behavior during courtship and according to sex-specific breeding cycle chronology (males being the first to incubate upon egg-laying [27]). Males (N = 102) were banded on laying-date and females (N = 89) some 15 days later, upon their return from the foraging trip at sea, to relieve their partner. Those birds were followed daily from a distance to determine their breeding status (incubation or brooding shift). A small fraction of the studied animals (N = 15) was marked when already incubating and neither sex nor shift were known.

### Time-Budget of Global and Specific Comfort Behaviors

#### Comfort behavior in penguins

Based on [5], we characterized six major types of comfort behaviors: preening, head-shaking, head-scratching, stretching, tail-wagging and yawning. Preening (re-arrangement of feathers and parasite removal on the breast, belly and flippers with the beak or head) and head shaking (brief lateral movements) were associated with moderate physical activity. Head scratching with a foot (which requires contorting and use of the tail to maintain balance) and stretching (full body stretch almost always associated with strong flipper flapping) corresponded to vigorous physical exercise. Yawning (head tilted backward, bill opened) and tail-wagging (sequences of 5–6 lateral wags in a row) required only slight physical activity. Preening and head-scratching were devoted to maintenance of the plumage (removal of dry foreign materials and ectoparasites, waterproofing, thermal insulation). Head-shaking allowed to keep the head dry under rainy weather and to eliminate excess fluid secretions from the nasal ducts of the salt-glands, whereas tail-wagging was used to remove foreign material, feces or water from the tail and cloacal region, especially on rainy days. Stretching and to a lesser extent yawning are suggested to play a role in increasing proprioceptive sensitivity and circulation in the muscles, thus maintaining functional musculature and preventing ankylosis [5]. In this study, the duration of episodes of these specific behaviors ranged from a few seconds (yawning, head-shaking, and tail-wagging) to several minutes (head-scratching or preening). Often, several of these behaviors were associated within the same comfort sequence, e.g. preening and head-scratching, yawning and tail-wagging, which lasted several minutes. Head-shaking often ended with head-bobbing and swallowing and could be followed by whole body shakes.

#### Global comfort behavior

The time spent in global comfort behavior was determined by instantaneous scan sampling [36]. In 2006–2007, throughout the entire breeding season (November–March), we estimated the time budgets of 182 marked birds (90 males, 79 females and 13 unsexed birds) not equipped with HR loggers (see below). Birds were located in different parts of the colony and were early or late breeders at different stages of breeding, i.e. different incubation and brooding shifts. We recorded behavioral activities of thirty of these birds every five minutes during at least six consecutive hours. The comfort category included every type of comfort behavior. Scans were performed at a distance of 10–50 m, using binocular and spotting scopes to avoid disturbance of the birds. Individuals observed during scans were located at least 4 m apart to maximize independence of their behavior relative to their neighbors. We balanced observations during all hours of daylight, from 0600 to 2000 hrs. Behavioral data were obtained from a total of 2270 scans spread over 25 days and totalizing 189 hrs of observations.

#### Specific comfort behaviors

The contribution of the different types of comfort behavior to the overall time spent in comfort behavior was determined from two hundred 15-min focal observations [36] during which at least one episode of comfort occurred (i.e. 50 hrs in total). These focal observations were obtained from one hundred of the above mentioned individuals. The six types of comfort behavior characterized above were considered and the proportion of time devoted to these different behaviors was determined for each focal observation. Focal observations were obtained from 58 males (n = 136) and 42 females (n = 64) with 1 to 4 observations per bird. As for scan samplings, focal observations were spread over the breeding season and performed in birds of different incubation and brooding shifts.

### Energy Cost of Global and Specific Comfort Behaviors

#### Heart rate and video monitoring

The energy cost of comfort behavior was estimated from the corresponding increase in heart rate (HR) above resting values (see below). The recording of HR provides a good means for estimating energy expenditure (EE) in field studies [37], as it is a relatively non-invasive technique (i.e. when using external HR loggers) that offers the possibility to monitor *in situ* EE with a fine time-resolution. Recent studies have investigated its use in fasting king penguins [34], [38]–[41], including in freely-living breeding birds [33]. Here, we used externally mounted HR-loggers (Polar® model RS800, Polar Electro Oy, Kempele, Finland) specially adapted for suitable use on king penguins, as previously described in [33]. Briefly, the system included two units: a sensor-transmitter (30–40 g) and a receiver/logger (30 g). After disinfection with iodine (Betadine®) and alcoholic antiseptic solutions, electrodes made from gold plated safety needles were inserted under the skin in the subcutaneous fat layer (at approximately 5 mm depth, and over a length of 1 cm). One electrode was placed at the height of the wing pit and the second one above the tail. The whole HR logger package was secured in a dorsal, midline position using Tesa® tape. We ensured that loggers and electrodes remained out of the animals' preening reach so that birds were never observed attempting to remove electrodes or HR loggers, nor did we observe any adverse effects of equipment on birds' health or behavior. As used, the HR-logger yielded HR values highly comparable to those measured with a stethoscope [33]. The sampling rate was set at 1 value per 5 sec, allowing for 45 hrs of continuous HR monitoring without any intervention on or close to the equipped animals. We equipped with HR-loggers a total of 24 birds (12 males, 10 females and 2 unsexed birds) at various phases of the breeding cycle (i.e. different incubation and brooding shifts), and their behavior was monitored by continuous video recording (using IR lighting during the night) as previously described in [30]. Equipment was performed in late afternoon and, to ensure that birds' HR and behavior was no longer affected by handling, only data obtained at least 6 hrs after equipment were considered.

#### From heart rate to energy expenditure

Energy expenditure was estimated from HR using equation *1a* (obtained from a mixed-model approach) in [33]: EE (J/min) = −387+36.4*HR (bpm) (F_1,133_ = 19.33, R^2^ = 0.85, P<0.0001). A validation test showed that EE predicted from HR using the above equation did not differ significantly from measured EE (t = 0.54, n = 30, P = 0.60) [33]. As we used a different group of individuals (selected at random) from that of [33] in order to estimate EE from new field HR values, it was important to account for errors associated with: (1) the scatter around the original regression line in [33] (i.e. EE on HR), and (2) the variability between penguins (both for the calibration group in [33] and for the birds in our study) (see [41]). Thus, error terms for our estimates of EE were conservatively calculated after equation *11* in [41], further adapted to account for one other source of uncertainty, i.e. in the relationship between body mass and total body energy used to calculate EE from body mass loss in [33]. The advantage of such an equation is that it is obtained from freely-incubating male and female king penguins (no gender difference), i.e. for a breeding status, a level of physical activity and a situation exactly the same as in the present study. Such pre-requisites are required for validly estimating EE from HR [37], [42]. Moreover, as stress might affect the oxygen consumption (VO2) – HR relationship in captive animals [33], [42], ultimately leading to an underestimation of EE [33], an equation obtained from free-living birds may be more appropriate for estimating energy costs in the wild (see [33], [42]). Nonetheless, we are also aware that using an equation calibrated on a larger time-scale (days) than the time-scale over which behaviors are monitored (i.e. min in the case of comfort behaviors) may be subject to criticism [42]. For this reason, we compared our estimates to estimates obtained using a finer time-scale calibrated equation for king penguins (equation *1* in [34]), albeit the latter was obtained from likely stressed animals, as those were held captive for respirometry purposes. Unfortunately, the errors associated to the latter estimates could not be calculated, as the original data set used to establish equation 1 [34] is required for their calculation but is unavailable (unpublished).

#### Energy cost of global comfort behavior

From the simultaneous recording of HR and behavior, we estimated the global energy cost of comfort behavior by comparing two different methods. The first estimate (estimate 1) was based on the determination of how the proportion of time spent into comfort behavior during 15 min focal observations explained the variability of average HR during these focal observations. Amongst the 24 equipped birds, 5 individuals (3 males and 2 females) were selected at random and their behavior and HR were considered over a 24 hr period starting at midnight. We divided the 24 hr period into consecutive periods of 15 min during which the total time spent in comfort activity was determined (ninety-six 15-min focal observations per bird, i.e. 480 observations in total). These 480 focal observations spread over 24 hrs allowed us to search for a potential day-night pattern in comfort behavior. Average HR and the proportion of total time spent in comfort behavior (whatever the behavior) were calculated for each focal. Then, the relationship between average HR and the time spent in comfort behavior was determined, the slope of this relationship yielding the first estimate of the global energy cost of comfort behavior after converting HR into EE. However, the error term associated with this estimate could not be validly calculated, as the reasoning made considers an increase in proportion (see Results). Indeed, whereas a 1% increase in the time spent in comfort will lead to a constant increase in HR (i.e. the slope of the relationship), the associated error itself depends on the initial and final HR values used (i.e. the last term of the calculation of the error [41], [43] 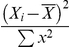, depends on X_i_, which is the heart rate at which we calculate the associated energy expenditure, see [41]). The errors would then not be the same when increasing the time spent in comfort behavior from 0% to 1% or from 5% to 6%, for example.

We thus established a second more conservative estimate (estimate 2) which was based on the determination of HR increase during selected episodes of continuous comfort behavior. These episodes had to fit two criteria: (1) they had to be preceded and followed by resting periods of at least 30 sec during which HR was stabilized at basal levels; and (2) only comfort behavior had to be performed during the considered episodes (very often comfort behavior is transitorily interrupted by episodes of aggressiveness related to territory defense). A total of ninety-four episodes over the 24 equipped birds were characterized, with 1 to 8 episodes per individual. The selected episodes were spread over 24 hrs and selection was at random concerning the type of comfort behavior so that data was considered representative of average comfort behavior. Only one type of comfort behavior was performed during half of the selected episodes whereas the other episodes were a mix of different types of comfort behavior. When obtained from the same individual, episodes were separated by at least two hours so that each episode was considered as independent. The average (± s.e) duration of the episodes was 2.3±0.5 min (from 10 sec to 28.6 min; n = 94). They were preceded and followed by resting periods averaging 2.2±0.3 and 2.6±0.3 min, respectively.

#### Energy cost of specific comfort behaviors

Based on the same method as for estimate 2 of the global energy cost of comfort behavior (and using the same 24 birds), the energy cost of specific comfort behaviors was calculated from selected episodes of comfort during which only one type of comfort behavior was performed. This was possible for five of the six types of comfort behavior that were characterized. Tail-wagging was almost always included into sequences of various comfort behaviors so that we did not succeed in selecting episodes of that behavior that were preceded and followed by a resting period. According to the type of behavior, the number of selected episodes ranged from 12 (head-scratching) to 31 (stretching), the average duration of episodes ranged from 0.1±0.0 (yawning, head-shaking) to 2.3±0.6 min (preening) and data were obtained from 5 to 13 individuals, with 1 to 11 episodes per individual. When obtained from the same individual, episodes were separated by at least two hours so that each episode was considered as independent.

#### Calculation of EE during comfort episodes

**Figure 1:**
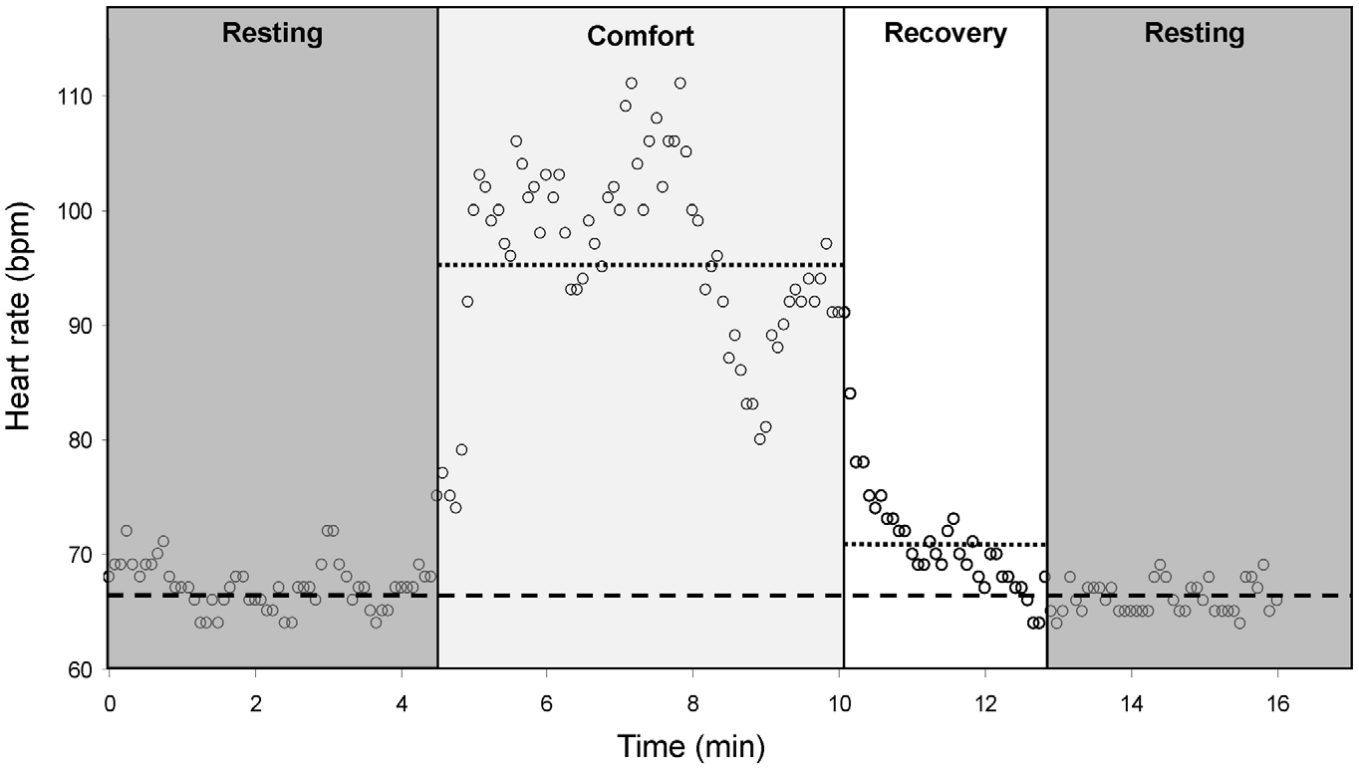
Heart rate increase during an episode of comfort behavior in an incubating king penguin. Shadowed zones delimit pre- and post-comfort resting periods and comfort behavior, respectively, whereas the white zone delimits the recovery period. Dotted lines give average HR during comfort behavior and recovery period, respectively, whereas the dashed lined gives average HR during pre- and post-comfort resting periods.

Energy expenditure during comfort episodes (both for estimate 2 of global comfort and for specific comfort behaviors) was determined according to [44] and as illustrated in Fig. 1. It was the energy spent in excess to resting metabolic rate (RMR) during comfort behavior plus the potential recovery phase. RMR was calculated from resting HR (mean of pre- and post-comfort resting HR). Comfort HR was the mean HR during comfort behaviors and recovery HR was the mean HR during the recovery phase. Comfort behaviors ended when the bird settled back into resting posture and the recovery phase ended when HR returned to resting levels. The recovery phase lasted on average 0.5±0.1 min (from 0 to 5.3 min, N = 192). Excess HR during comfort behaviors was calculated as [(comfort HR – resting HR)×comfort duration]/resting HR [44] and corresponded to the time that would be required for the number of heart beats in excess to occur at the resting HR level [45]. The same calculation was done for the recovery phase, using recovery HR, and the total excess due to a comfort episode was the sum of excess during comfort plus recovery. The energy cost of a comfort episode (kJ) was calculated as: excess in time (min)×RMR (kJ/hr). Dividing the cost of the episode by its duration (min) yielded an energy cost in kJ/hr.

### Contribution of Global Comfort Behavior to Daily Energy Expenditure

We calculated total daily energy expenditure (DEE), RMR and energy expended daily for activity for each of the 24 birds from which the energy cost of global comfort behavior was estimated (estimate 2). DEE and RMR were calculated from average daily and resting HR, respectively. Average daily HR of an individual was estimated from the HR measurements performed over the day (from 00h00 to 24h00) during which the episodes of comfort behavior were selected to calculate the average cost of comfort (estimate 2). Average resting HR of an individual was calculated from HR determination during the resting periods preceding and following the different comfort episodes selected on that day (2 to 16 resting HR estimates per bird). Energy expended daily for activity was calculated as DEE – RMR. The total energy expended daily for comfort behavior was obtained by multiplying the average energy cost of comfort by the average time spent daily into comfort behavior, as estimated from scan sampling. The contribution of specific comfort behaviors to the total energy cost of comfort was calculated by multiplying the proportion of comfort time spent in a specific behavior by its energy cost.

### Statistics

Statistical analyses were performed using R v.2.10.1 (http://www.r-project.org/) statistical software. To determine how the proportion of time spent in comfort behavior during 15 min focal observations explained the variability observed in average HR (estimate 1 of the cost of global comfort behavior), we ran a Generalized Estimating Equation (GEE, [46]) model in which the proportion of time spent in comfort behavior was entered as a dependant variable and individual and rank of the focal observation were set as random and repeated factors (first order autoregressive structure), respectively. This allowed us to control for repeated measurements as well as for individual (and thus sex) variability in HR. Similarly, day/night patterns in comfort behavior were checked by entering day/night as an independent factor variable in a GEE model, individuals (i.e. birds) as a random factor and the rank of focal observation as a repeated factor. Generalized estimating equations (GEE) were computed using the ‘geeglm’ function from the ‘geepack’ package in R v.2.10.1 [47] and marginal R^2^ was calculated according to [48]. When looking at differences between EE in different states (e.g. comfort *vs.* resting), errors were calculated using the method described page 682 in [41]. We calculated one EE value per bird and its associated error for each state (or behavior), then the difference in the estimate of EE between states/behaviors, and finally we averaged the difference over all birds (and calculated its associated error after equation *14*, in [41]). When comparing different behaviors (e.g. preening *vs.* stretching), we calculated the mean differences between those behaviors. The mean associated variance was then calculated as the sum of the variance associated with the two behaviors using equations *11* and *14* in [41]. A Z-statistic allowed us to test for significant differences. A simple approximate normal test was then used to look for differences between states. However, when looking for differences between specific behaviors, we preferred the use of a permutation test due to a lower sample size in some of our groups (i.e. n = 12 cases of head-scratching and n = 14 cases of head-shaking). The Z-statistic calculated when comparing two specific behaviors was then compared against the distribution of 1000 Z-statistics calculated from the values randomly redistributed between the two behaviors, and P-values for differences between specific behaviors were calculated accordingly. Significant results are reported for P<0.05 and Bonferroni's correction was applied whenever multiple comparisons were tested (differences were thus considered significant for P<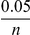 with *n* the number of comparisons done). Results are given as means ± standard error (s.e.) unless otherwise specified.

## Results

### Behavioral Time-Budget of Comfort Activities

Scan sampling data showed that king penguins breeding and fasting ashore spend on average 22.0±1.1% of time in global comfort behavior (n = 2270 scans). Focal observations (n = 200) showed that most of this time was devoted to preening (72.5±2.2%) and head-shaking (15.9±1.9%). Time spent in stretching and head-scratching was intermediate (3.7±0.4% and 4.0±0.6%, respectively), whereas only a limited amount of time was spent in tail-wagging or yawning (2.5±0.7% and 1.4±0.6%, respectively).

When considering data obtained from the five videoed birds of which comfort behavior was examined during consecutive periods of 15 min spread over 24 hrs, we found no apparent day/night pattern regarding the proportion of time spent in global comfort behavior (GEE; Wald = 0.07, P = 0.80, n = 480 focal observations).

### Energy Cost of Global Comfort Behavior

#### Estimate 1

**Figure 2:**
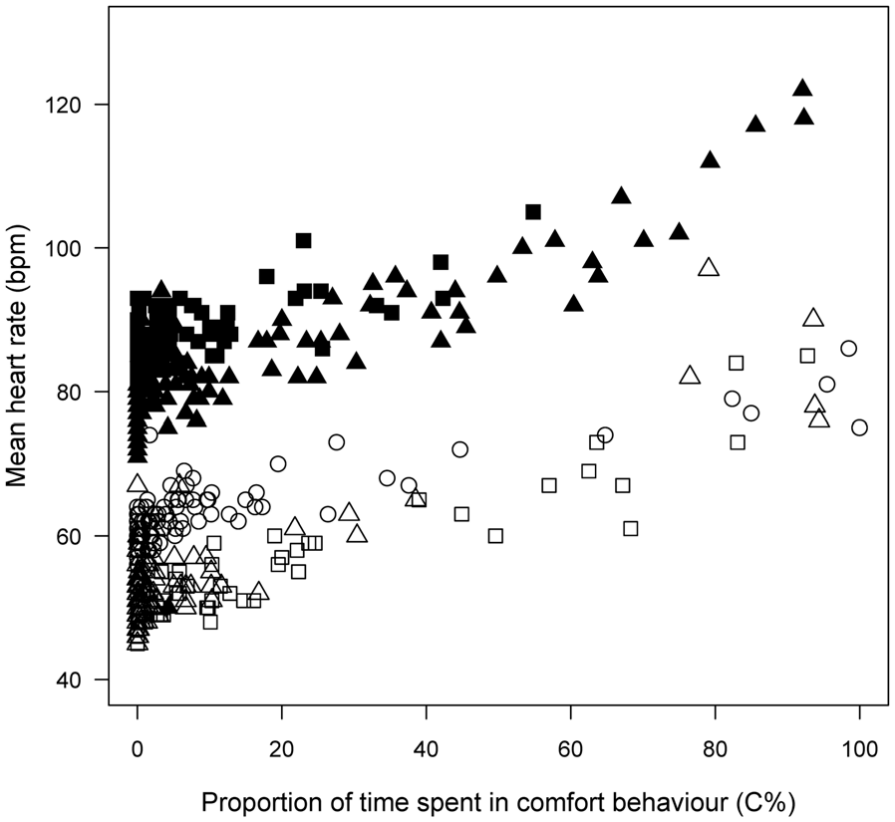
The energy cost of global comfort behavior in incubating king penguins. Relationships between the proportion of time spent in comfort behavior during 15 min periods (C %) and corresponding mean heart rate (HR) level. Data are for 3 males (□, Δ, ○) and 2 females (▪, ▴) with 96 values per individual. General equation for the mixed model is HR (bpm) = 65.89±3.95+0.28±0.02 * C %. (GEE, R^2^ = 0.19, Wald = 167, P<0.001). From the slope of the relationship and the equation relating HR to energy expenditure, the energy cost of comfort behavior was estimated at 61.2 kJ/hr (i.e. 17 W).

When included in the GEE model, the proportion of time spent in comfort behavior during 15-min focal observations significantly explained the variability of the corresponding average HR (R^2^ = 0.19, Wald = 167, P<0.0001, N = 5 birds, n = 480 focal observations). HR increased with proportion of time spent in comfort behavior (Fig. 2). From the slope of this equation (0.28) and from equation *1a* relating EE to HR (see above, [33]), we calculated that for a 1% increase in the time spent into comfort behavior (i.e. a 0.6 sec per min increase) the associated HR increase was equivalent to a 10.2 J increase in EE (36.4 * 0.28). Thus, the average energy cost of comfort behavior was 1.02 kJ/min, or 61.2 kJ/hr (i.e. 17 W).

Applying equation *1* in [34] to the same data, we found that a 1% increase in the time spent in comfort led to an 37.0 mL O_2_/min increase in oxygen consumption, equivalent to a 44.7 kJ/hr (i.e. 12.4 W) cost of comfort behavior (assuming the energy equivalent of 1 mL O_2_ is close to 20.112 J [49], [50]). As stated in the “Methods” section, a valid s.d. could not be calculated for these estimates.

#### Estimate 2

Resting HR averaged over the resting periods preceding and following the 94 selected comfort episodes was 67.8±1.4 bpm, whereas during comfort HR averaged 81.1±1.8 bpm. Applying equation *1a* in [33] to each individual HR values, we calculated that during comfort behavior EE averaged 3871±(s.d.) 334 kJ/day, whereas resting EE derived from resting HR values represented 3132±(s.d.) 297 kJ/day (i.e. a positive mean difference between comfort and resting states of 738±(s.d.) 336 kJ/day; Normal Test; Z = 2.19, P = 0.01). Hence, the energy expended during comfort behavior was 1.24 times RMR. From the total HR excess above resting HR, which accounts for both the increase in HR (including the recovery phase) associated to an episode of comfort and the duration of the episode, we calculated that the energy cost of global comfort averaged 58.2±(s.d.) 9.3 kJ/hr, or 16.2±(s.d.) 2.6 W (N = 24 birds, n = 94 episodes), i.e. a value differing from estimate 1 by only 5.0%. Applying equation *1* in [34] to these data, we found that average oxygen consumption during comfort behavior was 1.22 higher than when the animals were resting (i.e. 102.6 mL O_2_/min *vs.* 84.8 mL O_2_/min, for comfort and resting states, respectively). The average cost of comfort behavior was then 37.3 mL O_2_/min, corresponding to 45.0 kJ/hr (or 12.5 W), i.e. a value differing from estimate 1 by less than 1.0%.

### Energy Cost of Specific Comfort Behaviors

**Figure 3:**
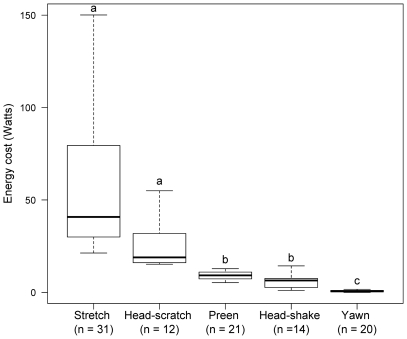
The energy cost of specific comfort behaviors in breeding king penguins. Light bars and dashed lines show the range of values, heavy bars medians. Boxes give the inter-quartile values. Sample sizes are given in brackets. Values not sharing a common superscript are significantly different for P<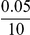.

During stretching and head-scratching, HR markedly increased above resting values, from 63.3±1.1 bpm to 106.3±3.1 bpm (n = 31), and from 63.7±2.8 bpm to 102.4±8.3 bpm (n = 12), respectively (Wilcoxon's test, W = 2, P<0.001 and W = 7, P<0.001, respectively). The average HR increase associated with preening (from 60.9±1.9 bpm to 75.4±2.2 bpm, n = 21) and head-shaking (from 63.4±2.3 bpm to 75.2±3.8 bpm, n = 14) was moderate (Student's t-test, t = −4.875, P<0.001 and t = −2.632, P = 0.015, respectively) whereas no significant HR increase was observed during yawning (Wilcoxon's test, W = 168.5, P = 0.40, n = 20). Correspondingly, energy expenditure calculated from equation *1a* in [33] ranged from 1.03 (i.e. yawning) to 1.78 (stretching) times RMR. From the total HR excess above resting HR (including the recovery phase) associated to specific behaviors, we calculated that the energy cost of stretching was 2, 6, 9 and 61 times more than that of head-scratching, preening, head-shaking and yawning, respectively (Fig. 3). Similar results were obtained when using equation *1* in [34]. For example, cost of stretching was roughly 2, 6, 9 and 60 times more than that of head-scratching, preening, head-shaking and yawning, respectively. From these costs and from the proportion of comfort time spent in the different types of comfort behavior, we estimated that approximately half of the energy cost of comfort behavior was for plumage cares (preening plus head-scratching).

### Contribution of Comfort Behavior to Daily Energy Expenditure

The daily energy cost of comfort behavior calculated by multiplying the time spent each day in comfort behavior (22% or 5.28 hr) by its cost was 323 kJ/day (estimate 1), 307 kJ/day (estimate 2). The average daily and resting HR of the 24 birds used to determined DEE, RMR and the average cost of comfort behaviors (estimate 2) were 73.4±3.0 and 65.0±2.6 bpm, respectively. The corresponding DEE and RMR were 3465±(s.d.) 306 and 3026±(s.d.) 296 kJ/day, respectively. The energy cost of comfort behavior thus represented 9.3–8.8% of DEE (estimates 1 and 2, respectively). The cost of activity, i.e. the difference between DEE and RMR, was 440 kJ/day (or 12.7% of DEE), and most of this cost corresponded to comfort activities (*viz.* 73.5–69.8%; estimates 1 and 2, respectively). When using equation *1* from [34], the average daily energy cost of comfort behavior, the average DEE and RMR were estimated at 238, 2748 and 2416 kJ/day, respectively. Thus, consistently with the results presented above, the energy cost of comfort behavior and of activity calculated using equation *1* in [34] represented 8.6% and 12.1% of DEE, respectively.

## Discussion

To our knowledge, this study is the first to estimate the contribution of comfort behavior to overall energy budget in a free-living animal. It shows that, although fasting for a prolonged duration, king penguins devote a substantial part of their daily time and energy budget to comfort behavior when breeding ashore. Approximately half of the energy cost of comfort was allocated to plumage maintenance and half to behaviors involving a vigorous muscular activity. These results highlight the importance of comfort behavior for self-maintenance in colonial seabirds and suggest that the energy invested into comfort activities is the necessary price to pay for animals to maintain a good body condition and proper physical fitness, potentially at the expense of immediate breeding success but to the benefit of survival and foraging efficiency. Before discussing the adaptive significance of our results, it was necessary to consider whether the methodology used yielded valid estimates of both time-budget and energy expenditure.

### Comfort Time and Energy Budget

We found that incubating penguins spend 22% of their daily time budget in comfort behavior. This figure was obtained using a very large number of birds and instantaneous scan sampling equally spread over the whole breeding season, including birds of both sexes and all breeding states representative of the successive incubation and brooding shifts observed in king penguins. Importantly, this figure was derived from wild birds that were not equipped with HR loggers; so that time spent preening could not have been influenced by the attachment of external devices to the animals' body (see [51]). Additionally, and in agreement with a previous report [32], we obtained evidence that there was no day/night pattern in comfort behavior. Thus, we are confident that the 22% figure obtained here for the overall proportion of time in comfort behavior is representative of king penguins breeding ashore, over 24 hrs. In a previous study, this proportion was estimated at 16% [32]. However, since these data were obtained from a limited number of individuals observed for only one day, they may not be fully representative of the whole population throughout the whole breeding season.

When estimating EE from HR, one should be cautious with the calibration equation used. The method of calibration needs to match the data that are being estimated as closely as possible to avoid potential increases in the error associated to the prediction made [42]. The question is then whether equations calibrated over longer time scales (i.e. equation *1a* in [33], calibrated over 4 days) are appropriate for estimating the energy cost of behaviours that last only minutes? Whereas this concern is most certainly justified, one should bear in mind the trade-off scientists must face. Establishing calibration equations relating HR to EE over the scale of seconds or minutes is simply not possible unless it is done by measuring oxygen consumption (VO_2_), which then requires keeping animals captive and monitored in respirometry chambers. Although this approach is undoubtedly the most thorough, it raises the issue of experienced stress [33], [42], which may well influence the HR–VO_2_ relationship (as HR is then not necessarily entirely reflective of actual oxygen uptake), leading to biases in the estimation of EE [33]. This may be the case in our study where we observe that when using equation *1* from [34], which was calibrated over a period of time closer to the duration of comfort behaviors, we find estimates of EE for global and specific comfort behavior to be 20 to 27% lower than estimates of EE calculated from equation *1a* in [33], which was derived from wild birds fasting, incubating and having a moderate level of physical activity (i.e. animals in a situation similar to those used in this study). The discrepancy observed between the estimates is consistent with that previously reported in [33]. Nonetheless, as the difference is constant, it is reassuring to find that regardless of the equation used, the cost of global comfort behavior when expressed as a proportion of RMR was almost identical (1.22 or 1.24×RMR, for estimate 2 for instance). Also, using both equations from [34] and [33], we found similar relative costs of specific comfort behaviors as well as similar contributions of comfort behavior to daily energy expenditure (e.g., 8.6–9.3% of DEE, respectively). Such findings support the view that EE *vs.* HR equations obtained by using two markedly different methods may yield different levels of overall energy expenditure but similar relative costs of specific activities, in this case comfort behavior.

Further, the validity of our estimates is also supported by considering our results on the contribution of comfort behavior to energy expended for total activity, and previous studies. Based on HR recording over 24 hrs, we calculated that total activity contributed for 12.7% to DEE. In breeding king penguins, active behaviors include comfort behavior, aggressive behavior and parental care provided to the egg or chick, the two later behaviors representing 8–24% and 1–2% of the time budget [32]. Aggressive behaviours contribute for 2.7% to DEE [52]. The contribution of comfort and aggressive behaviors (by far most frequent active behaviors) to DEE summed up to 11.3–12.0%, depending on whether equation in [33] or [34] is used. This sum is very close to the contribution of all daily activities to DEE (i.e. 12.1–12.7% according to which equation is used) suggesting that the cost of comfort determined in the present study is realistic. The energy cost of egg and chick caring (which involve movements with an intensity comparable to that of comfort and aggressive behaviors but only contribute to 1–2% of time-budget) would be very minor (<0.5% of DEE), which also seems reasonable. Additionally, the finding that stretching and head-scratching are more costly than preening, head-shaking and yawning was to be expected, given that the former are associated with vigorous muscular activity whereas the latter only require discrete activity.

The proportion of time devoted to comfort behavior by king penguins (22%) is amongst the highest reported for birds [20]. It is also higher than the proportion of time spent in territory defense (8–10% [32], 11.5–18.7% [53]), a behavior that appears highly beneficial to breeding success in this colonial species (Viera, Côté and Groscolas, unpublished data). In addition, previous data (Viera, Groscolas and Côté, unpublished data) indicated that there was no difference in the time invested into comfort according to gender or to some parameters affecting breeding success. Actually, the time spent in comfort does not differ between birds located at the periphery *vs.* the centre of the colony, although breeding success is suggested to be higher at the centre [53]. Similarly, early and late breeding birds devoted the same proportion of time to comfort, even though breeding success is markedly lower in late breeders [28]. Lastly, [32] reported that *i.* the time devoted to comfort behavior was similar in incubating and brooding penguins, and *ii.* the same proportion of time was spent for comfort behavior throughout an incubation shift, *i.e.* whatever the fasting duration and thus energy stores. Thus, it appears that king penguins spend a high proportion of time in comfort behavior regardless of energy constraints imposed by their breeding pattern, and regardless of some components of their breeding success. If comfort behavior were not energy costly, this high proportion of time could be the mere consequence of the fact that king penguins breeding ashore have no major time constraints (e.g. for food searching or anti-predator defense) and thus may devote a large part of time to other behavior, e.g. body maintenance. Actually, devoting approximately 9% of its energy budget to comfort behavior while totally depending on energy reserves for surviving must be considered as costly. Indeed, this cost is equivalent to the energy required to fuel DEE for about 1.5 of the 15 day incubation shift. When energy reserves are close to exhaustion, being able, or not, to fast for 1.5 supplementary days while waiting for the return of the partner might well mean going on incubating or abandoning the egg, i.e. being a successful breeder or not. Thus, the energy expended for comfort behavior might be at the expense of immediate breeding success. On the other hand, the finding that king penguins are willing to pay a substantial energy cost for comfort behavior strongly supports the view that this behavior is adaptive and procures major benefits, including from an energy view point.

### Adaptive Significance of Comfort Behavior

In incubating penguins, comfort behavior likely plays an essential role in the maintenance of a functional outer shell and musculature, and in the removal of ectoparasites. Given that they are fasting and must spare energy [29], [54], incubating king penguins can not afford the potential excess energy costs associated with a decrease in the insulating and waterproofing properties of their plumage. The same is of even greater importance for penguins swimming and diving into cold waters when intensively foraging between two incubation shifts ashore. Unfortunately, no data are available to estimate how much defects in plumage integrity may incur thermal costs in penguins, and thus how much maintenance of plumage through preening may allow energy saving. A doubling in body mass loss reflecting a comparable increase in metabolic rate has been observed in molting and thus poorly insulated penguins fasting ashore [55]. However, how this increase partitions between thermal loss and feather synthesis is unknown. On the other hand, data on the energy cost of ectoparasite loads have been obtained both in birds and mammals. For example, a high bug (*Oeciacus hirundinis*) load imposes an about 13% increase in mass independent DEE in house martin (*Delichon urbica*) nestlings [24]. In the feral dove (*Columbia livia*), a high load in feather-feeding lice (Phthiraptera: Ischnocera) reduced feather mass, leading to an 8–12% increase in thermal conductance and to a 10% increase in basal metabolic rate [12]. Lastly, in mouse-eared bats (*Myotis myotis*), a high mite (*Spinturnix myoti*) load induces an up to 21% increase in O_2_ consumption and a 15% body mass loss compared to non-infested individuals [2]. Thus, a high ectoparasite load may incur significant energy costs, in addition to other negative effects such as inoculation of toxins and transmission of pathogens. King penguins are known to be infested by various species of ectoparasites, including *Ixodes uriae* ticks known to be a vector of viruses and of the Lyme disease agent *Borrelia burgdorferi* [56]. In the study colony, a reduced incubating success has been observed in infested areas [57] and hyperinfestation by ticks has been suggested as a possible cause of death in adults [56]. Ensuring thermal insulation, by keeping the plumage in a good condition in one of the windiest and rainiest places on earth, and limiting ectoparasite load, may well be essential for king penguin survival. Thus, it is understandable that this bird devotes the greatest part of time and about half of the cost of comfort behavior (i.e. roughly 5% of DEE) to preening activities.

Besides the need to have a perfectly insulated and waterproof plumage, ashore and at sea, and to limit ectoparasite load, king penguins would probably take advantage of being efficient foragers as soon as they return to sea to replenish their energy stores. This would allow them to limit the duration of their foraging trips at sea, and thus to limit the risk of egg desertion by their incubating partner. Given that they use feeding grounds situated several hundreds of kilometers from the colony [58], [59], and that they have to dive repeatedly at great depths (over 200 m) to catch their prey [60], [61], it would be advantageous for king penguins departing to sea after an incubation shift to be as physically fit as possible. Maintaining minimum levels of muscular activity and preventing muscular ankylosis while on land (through shaking, stretching or other vigorous comfort activities), even if it costs around 5% of DEE, might well be a necessary condition for penguins to maintain this physical fitness. We therefore suggest that the energy invested in comfort behavior by breeding king penguins is the necessary debt to be paid ashore in order to maintain plumage insulation and waterproofing, to limit the impact of ectoparasitism, and to be optimal divers and foragers when they return at sea. Such an energy investment may contribute to improve penguin survival and foraging efficiency.

These suggestions are mostly based on the determination of the average cost of comfort behavior over a breeding season and for penguins located in a given part of the colony. A full understanding of the adaptive significance of comfort behavior, and, more generally, of how energy and environmental constraints shape the behavioral repertoire of colonial seabirds, will obviously require further investigations. First, it would be interesting to determine whether the time and energy devoted to preening is actually fixed or rather related to parasite load. This could be achieved by comparing the time invested into preening at different locations of the study colony known to have different parasite loads [57], and by examining the relationship between preening time and parasite load at the individual level. Second, examining whether comfort behavior competes with other behaviors such as territory defense will allow a better understanding of how colonial birds trade time and energy between self-maintenance and behaviors more directly related to reproductive success. Indeed, visual observations of king penguins indicate that engaging into comfort behavior very often induces aggressiveness from neighbors such as these two behaviors seem at least partly conflicting. Lastly, whether comfort activities involving vigorous physical motions (e.g. stretching, shaking) may contribute to maintain physical fitness thus improving foraging efficiency could be tested by relating the time devoted to these specific behaviors while breeding on land to swimming, diving and foraging performances at sea. This would help understanding how, in seabirds, behaviors ashore and at sea are energetically interrelated.
